# The Impact of Recurrent Epistaxis on the Quality of Life of Children and the Functioning of Their Families

**DOI:** 10.7759/cureus.57324

**Published:** 2024-03-31

**Authors:** Raed A Alfayez, Abdullah Alhashim, Mohammed Alkhars, Rawan Y Bonayan, Mohammed A Alnahwi, Abdullah Alarfaj, Khalid Alyahya

**Affiliations:** 1 Department of Surgery, College of Medicine, King Faisal University, Alahsa, SAU; 2 Department of Surgery, Otolaryngology Unit, College of Medicine, King Faisal University, Alahsa, SAU

**Keywords:** child health, parental stress, family dynamics, quality of life, pediatric epistaxis

## Abstract

Introduction

Epistaxis, or nosebleeds, is a common pediatric emergency, impacting their quality of life (QoL). Existing research on epistaxis has predominantly focused on clinical aspects, overlooking its broader impact on the quality of life of affected children and the functioning of their families. This study seeks to fill that gap by assessing the impact of recurrent epistaxis on children's QoL, family dynamics, and parental stress in Saudi Arabia's Eastern region.

Methods

A survey was conducted involving 168 parents of children with recurrent epistaxis, using the Pediatric Quality of Life Inventory^TM^ (PedsQL 4.0^TM^) Short Form (SF) for QoL assessment across different age groups, the PedsQL 2.0 Family Impact Module to evaluate the effect of the child's health on family dynamics, and a custom questionnaire for gathering sociodemographic and health-related information. Better QoL and family functioning were indicated by higher scores.

Results

Recurrent epistaxis was more frequent (>4 times per year) in 58.9% of cases, with unknown causes in 72%. A total of 116 (69%) of the children never needed medical intervention for epistaxis and 52 (31%) visited ER 1-2 times. The lowest scores for both children and parents were in the emotional functioning domains (77.9 and 78.2, respectively). In the study, both parents and children who had no history of ER visits exhibited significantly higher quality of life (QoL) scores compared to those who did, with parents reporting 83.7% versus 77.2% (P=.022), and children showing 84.6% versus 79.9% (P=.049), respectively. Parents of older children, ages 13-18 years, reported a higher Quality of Life (QoL) at 83.9%, compared to those with younger children, ages 2-4 years, who reported a QoL of 57.3% (P=.003).

Conclusion

The overall QoL scores of families of children with recurrent epistaxis were relatively high, indicating a variable and limited general impact. Significantly higher QoL was observed in families of older children and those without ER visits.

## Introduction

Epistaxis is a frequently encountered issue among pediatric patients, yet its precise prevalence remains uncertain. Studies indicate a wide range of prevalence, with approximately 60% of cases occurring in the population, of which only 6% seek medical attention [1]. By the age of 10, more than half of children have experienced epistaxis, accounting for approximately one in 260 visits to the emergency department among children below 19 years old in the United States [2]. In Saudi Arabia, the prevalence of epistaxis in the pediatric population is not well-documented. However, one study showed a prevalence of around 27% among young adults [3]. Recurrent epistaxis lacks a consensus definition regarding the duration or frequency of episodes, although some studies consider it as five or more episodes per year [4]. The majority of pediatric epistaxis cases originate from the anterior septum and typically resolve spontaneously. Causes such as posterior bleeding sites, anticoagulant use, and hypertension are rare in children, while digital trauma, vestibulitis, and crusting are more commonly observed [5,6]. Although severe cases necessitating nasal packing or hospitalization are rare among children, as most episodes are spontaneous, anterior, and self-limiting, it can affect the quality of life of the families concerned as epistaxis often makes patients feel a loss of control due to limited influence over the duration and frequency of episodes [7,8]. Only two studies thus far measured the impact of recurrent epistaxis on the families or parents concerned [7,9]. Our study aims to determine the impact of recurrent epistaxis on family functioning and its impact on children's quality of life in the parents' view.

## Materials and methods

A cross-sectional observational study that was conducted from August to October 2023 in the eastern province of Saudi Arabia, which includes the following areas: Al-Ahsa, Dammam, Al-Khobar, Dhahran, Al-Jubail, Hafr Albatin, and Al-Qatif. Parents of children aged 2 to 18 years, residing in the specified areas and experiencing epistaxis three or more times per year were included in the study if they consented to participate. Subjects aged under two or more than 18 years and those living outside the eastern province were excluded. An online questionnaire was created in Arabic using Google Forms and distributed on Social Media platforms (X, Telegram, and WhatsApp), involving the consent request before study participation. Ethical clearance was taken from the Institutional Review Board (IRB) at King Faisal University, Reg No KFU-REC-2023-JUN-ETHICS1034.

Demographic data 

Parents were asked to complete a questionnaire regarding demographic data, which was divided into two sections: one for the parents and one for their child. It included their relationship to the child, the parent's age, place of residency, and the parent's educational level. It also included their children's demographic information, including their gender, age, the frequency of epistaxis attacks, emergency department visits and management, and the possible causes. 

Evaluation of quality of life 

The impact of epistaxis on the quality of life of the parents and children involved was assessed using standardized questionnaires, including the Pediatric Quality of Life Inventory (PedsQL^TM^) 4.0 to assess children's Quality of Life across various age categories [10-12], and the PedsQL 2.0 Family Impact Module to evaluate the effects of a child’s chronic health condition on family functioning [13-15]. The Arabic version of all instruments was used. The PedsQL questionnaire, designed to measure Quality of Life in children with acute and chronic diseases, along with the PedsQL Family Impact Module, is recognized for its strong reliability, validity, and sensitivity, with internal consistency reliabilities surpassing the minimum alpha coefficient standard of 0.7 [10-15]. The PedsQL Short Form (SF15) 4.0 Generic Core Scales evaluates four areas: physical, emotional, social, and school functioning. The tool generates two composite scores: a psychosocial health summary and a total QoL score. PedsQL 2.0 Family Impact Module evaluates the impact of a child's health on family functioning across eight areas, including physical, emotional, and social aspects, among others. It calculates three summary scores: one reflecting the parents' health-related quality of life, another assessing overall family functioning, and a total impact score encompassing all eight areas. These scores are derived by averaging relevant items, providing a comprehensive view of the family's well-being in relation to the child's health condition.

Statistical analysis 

After data were extracted, it was revised, coded, and fed to statistical software SPSS version 22 (IBM Corp., Armonk, USA). All statistical analysis was done using two-tailed tests. A P-value of less than 0.05 was statistically significant. Regarding children and parents' quality of life, PedsQL discrete items scores were transformed into a 0-100 scale, with the mean score for each domain obtained by summing the items over the number of items answered. While there is no standard classification for the scores, the scores allow for comparative analysis, where higher scores indicate better functioning in the respective domains [12]. Descriptive analysis based on frequency and percent distribution was done for all variable participants' demographic data, epistaxis clinical patterns, and needed interventions. Also, children's and their parents' quality of life was presented in a range with mean and standard deviation. Cross tabulation was used to assess factors associated with children and their parents' quality of life, and the significance of relations was tested using One Way ANOVA and independent samples t-test. A correlation diagram (scatter plot) was used to assess the nature and strength of the association between children and their parents' QoL scores. 

## Results

A total of 168 eligible parents completed the study questionnaire. Exactly 111 (66.1%) of these parents were mothers of the children. The ages of the parents ranged from 18 to over 55 years, with a mean age of 37.5 ± 11.4 years. A total of 108 parents (64.3%) were university graduates, 82 (48.8%) had a monthly income exceeding 10,000 Saudi Riyal (SR), and 68 (40.5%) had a monthly income of 5,000-10,000 SR. The ages of the children ranged from 2 to 18 years, with a mean age of 9.6 ± 3.1 years. A total of 105 children (62.5%) with recurrent epistaxis were males (Table 1).

**Table 1:** Parents and children with recurrent epistaxis personal characteristics in Eastern Region, Saudi Arabia SR: Saudi Riyal

Parents / child data	No	%
Respondent		
Mother	111	66.1%
Father	57	33.9%
Age in years		
18-24	10	6.0%
25-34	35	20.8%
35-44	53	31.5%
45-54	46	27.4%
55+	24	14.3%
Education		
Below secondary	12	7.1%
Secondary	48	28.6%
University / above	108	64.3%
Monthly income		
< 5000 SR	18	10.7%
5000-10000 SR	68	40.5%
> 10000 SR	82	48.8%
Child age		
2-4	6	3.6%
5-7	43	25.6%
8-12	65	38.7%
13-18	54	32.1%
Child gender		
Male	105	62.5%
Female	63	37.5%

The clinical pattern of recurrent epistaxis among children in the eastern region of Saudi Arabia was assessed. Among the study participants, 99 (58.9%) children experienced epistaxis more than four times per year. The cause of epistaxis remained unknown in the majority of cases, involving 121 children (72%). Nasal allergy was reported as the cause in 29 cases (17.3%), while seven children (4.2%) were reported to have a hematological disorder as the underlying cause.

A total of 116 children (69%) never required medical intervention for epistaxis, while 52 (31%) visited the emergency room 1-2 times. Hospitalization for epistaxis was necessary for only seven children (4.2%). The most common interventions included cauterization (32 cases, 19%), nasal packing (18 cases, 10.7%), and embolization (five cases, 3%). Interestingly, the majority of children (70.2%) did not require any intervention to control nasal bleeding (Table 2).

**Table 2:** Clinical pattern of recurrent epistaxis among study children, Eastern Region, Saudi Arabia

Epistaxis profile	No	%
Epistaxis episodes per year		
3-4 times	69	41.1%
> 4 times	99	58.9%
Causes of recurrent epistaxis		
Unknown	121	72.0%
Nasal allergy	29	17.3%
Hematological disorder	7	4.2%
Others	11	6.5%
Frequency of visit ED per year due to epistaxis		
Never	116	69.0%
1-2 times	52	31.0%
Previously hospitalized due to epistaxis		
Yes	7	4.2%
No	161	95.8%
Interventions to stop epistaxis		
No intervention done	118	70.2%
Cauterization	32	19.0%
Nasal packing	18	10.7%
Embolization	5	3.0%
Others	6	3.6%

The impact of children's recurrent epistaxis on family functioning was assessed. The highest mean score was for communication (84.7%), followed by social functioning (83.5%), cognitive functioning (82.8%), worry (80.7%), physical functioning (78.7%), and emotional functioning (78.2%). For daily activities and family relationships, low effects were reported with mean scores of 82.2% and 85.2%, respectively. The overall impact score for parents was 81.7% (Table 3).

**Table 3:** The impact of children recurrent epistaxis on the family functioning, Eastern Region, Saudi Arabia QoL: quality of life

QoL domain	Range	Mean^#^	SD	Interpretation ^
Physical functioning	0-100	78.7	21.2	High
Emotional functioning	25-100	78.2	22.2	High
Social functioning	12.5-100	83.5	20.5	High
Cognitive functioning	0-100	82.8	21.2	High
Communication	25-100	84.7	20.8	High
Worry	10-100	80.7	21.8	High
Daily activities	0-100	82.2	23.5	High
Family relationships	0-100	85.2	21.0	High
Overall QoL	30.6-100	81.7	17.2	High

Quality of life among children with recurrent epistaxis was evaluated. The highest mean score was for Social functioning (87.5%), followed by School functioning (85.2%), Physical functioning (82.8%), and Emotional functioning (77.9%). The overall QoL score for children was 83.1% (Table 4).

**Table 4:** Quality of life (QoL) among children with recurrent epistaxis, Eastern Region, Saudi Arabia

QoL domain	Range	Mean^#^	SD	Interpretation ^
Physical functioning of child	0-100	82.8	21.2	High
Emotional functioning of child	6.3-100	77.9	22.2	High
Social functioning of child	0-100	87.5	20.5	High
School functioning of child	16.7-100	85.2	21.8	High
Overall child QoL	35-100	83.1	15.1	High

Factors associated with recurrent epistaxis of children on their parents' QoL were examined. A significantly higher QoL score was reported among fathers than mothers (85.7% vs. 79.6%, respectively; P=.028). Additionally, higher QoL was reported among parents with children aged 13-18 years (83.9%) compared to others with children aged 2-4 years (57.3%; P=.003). Likewise, parents of children who never visited the ER had higher QoL than others (83.7% vs. 77.2%; P=.022) (Table 5).

**Table 5:** Factors associated with recurrent epistaxis of children on their parents QoL (impact score) P: One Way ANOVA; ^: Independent t-test; P < 0.05 (significant); QoL: quality of life; SR: Saudi Riyal

Factors	Overall parent QoL		p-value
	Mean	SD	
Respondent			t=2.2 P=.028*
Mother	79.6	16.8	
Father	85.7	17.3	
Age in years			F=2.9 P=0.161
18-24	74.2	22.1	
25-34	78.6	17.6	
35-44	80.5	16.8	
45-54	86.1	15.8	
55+	83.5	16.7	
Education			F=1.9 P=0.616
Below secondary	86.4	16.6	
Secondary	81.1	16.1	
University / above	81.4	17.7	
Monthly income			F=2.2 P=.312
< 5000 SR	81.9	18.5	
5000-10000 SR	79.3	16.8	
> 10000 SR	83.6	17.2	
Child age			F=7.8 P=.003*
2-4	57.3	12.3	
5-7	80.7	16.0	
8-12	82.7	17.4	
13-18	83.9	16.5	
Child gender			t=1.4 P=.283
Male	82.8	17.0	
Female	79.9	17.5	
Epistaxis episodes per year			t=0.96 P=.970
3-4 times	81.8	17.2	
> 4 times	81.6	17.3	
Causes of recurrent epistaxis			t=1.79 P=.127
Unknown	83.0	16.5	
Nasal allergy	78.4	18.6	
Frequency of visit ED per year due to epistaxis			t=2.11 P=.022*^
Never	83.7	16.8	
1-2 times	77.2	17.3	
Previously hospitalized due to epistaxis			t=1.87 P=.178^
Yes	73.1	19.4	
No	82.1	17.0	

Factors associated with the impact of recurrent epistaxis on children's quality of life varied by age. Children aged 5-7 years had the highest QoL at 87.7%, while those aged 8-12 years exhibited the lowest QoL at 79.9% (P=.047). Additionally, children who never required an ER visit demonstrated significantly higher QoL compared to those who did (84.6% vs. 79.9%, respectively; P=.049) (Table 6).

**Table 6:** Factors associated with the impact of recurrent epistaxis on children's quality of life P: One Way ANOVA; *P < 0.05 (significant); QoL: quality of life

Child factors	Overall child QoL		p-value
	Mean	SD	
Child age			F=4.2 P=.047*
2-4	86.9	13.1	
5-7	87.7	10.4	
8-12	79.9	16.1	
13-18	82.9	16.4	
Child gender			t=1.12 P=.876
Male	83.0	14.9	
Female	83.4	15.5	
Epistaxis episodes per year			F=3.1 P=.190
3-4 times	85.0	14.5	
> 4 times	81.9	15.3	
Causes of recurrent epistaxis			t=1.3 P=.607
Unknown	83.5	14.9	
Nasal allergy	82.2	15.5	
Frequency of visits ED per year due to epistaxis			t=2.1 P=.049*
Never	84.6	14.4	
1-2 times	79.9	16.0	
Previously hospitalized due to epistaxis			t=1.6 P=.466
Yes	79.0	22.3	
No	83.3	14.7	
Needed intervention to stop bleeding			t=1.8 P=.166
Yes	80.7	14.0	
No	84.2	15.4	

**Figure 1:**
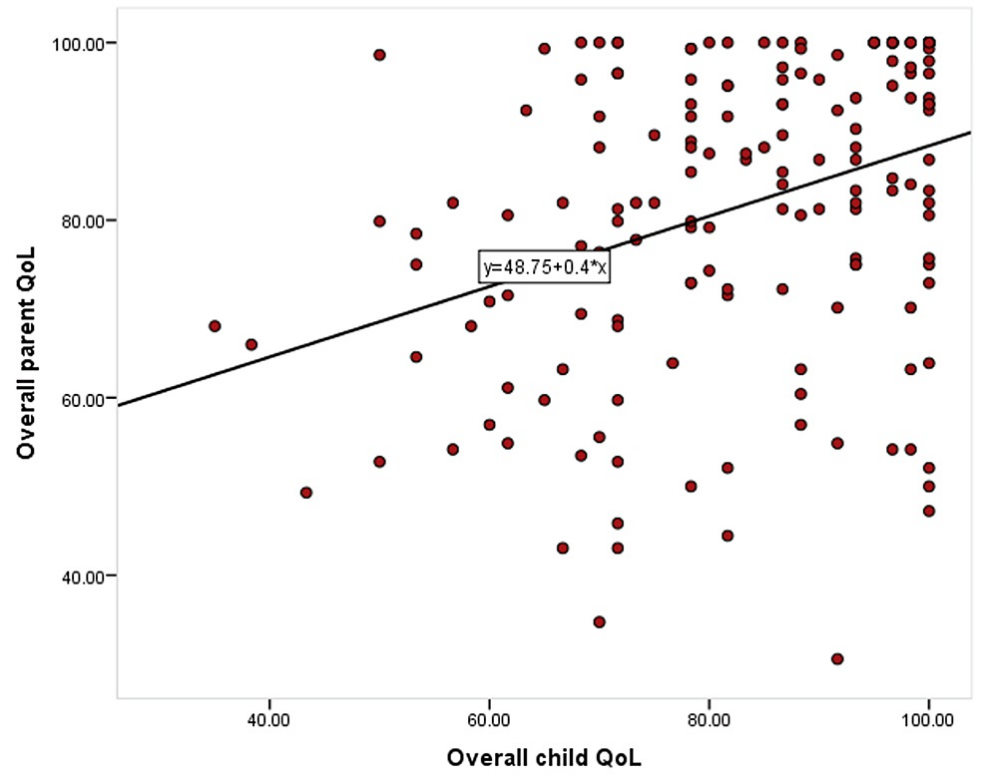
Scatter plot for correlation between the quality of life (QoL) of children with recurrent epistaxis and their mother's quality of life (r=0.35; P=0.001)

Regarding the correlation between children and their parents' QoL, there was a significant weak positive correlation between children's QoL and their mother's QoL scores, which means higher child QoL was associated with higher parents' QoL (Figure 1).

## Discussion

Epistaxis in children is a frequent problem faced by primary care physicians and otolaryngologists [16]. Epistaxis usually occurs between the ages of 3 and 8. The incidence of epistaxis decreases in adulthood, but approximately 50% of all adults who experience nosebleeds have had them during childhood [17]. Children under two years rarely suffer from nosebleeds (1 in 10,000 cases). In such cases, trauma (whether accidental or non-accidental) or serious illnesses like acute leukemia should be suspected [18]. No agreement exists on the frequency or duration of episodes that define recurrent epistaxis. However, some studies have defined it as five or more episodes per year [19].

The current study aimed to assess the impact of recurrent epistaxis on the quality of life of children and family functioning. The study showed that most of the children experienced recurrent epistaxis aged 8-18 years and were males. This may be associated with higher outdoor activity, mainly among males. More than half of the children had episodes of epistaxis more than four times per year, mainly due to unknown causes. These episodes were not severe as more than two-thirds of the children never visited the ED for epistaxis, and very few percent needed hospitalization, and this explains why about three-fourths did not need any medical intervention. Recurrent idiopathic epistaxis is self-limited nasal bleeding with no identifiable cause. It affects up to 9% of children, and only severe cases are treated, which is in accordance with the findings of the current study [20].

The ages reported among affected cases ranged from 11-14 years, similar to the most reported ages among the children in the current study [4]. Considering children's quality of life, the current study revealed high scores for all domains, mainly social functioning, school functioning, physical functioning, and emotional functioning, where all children reported an average score exceeding 75 out of 100. In total, the overall QoL score for children was 83.1%. A similar study in Egypt reported that the most affected domains among children with epistaxis were physical activities, as 36.6% complained of pain, and 29.7% had lethargy [21].

Recurrent epistaxis can have a significant impact on the quality of life of the affected family. One study found that the children affected by daily bleeds reported higher total stress scores secondary to the disruptive effect on school and classroom activities [7]. Another study also showed that 34% of cases had daily episodes, and 45% of patients had at least weekly bleeds, reiterating recurrent epistaxis as a significant problem [22]. In a study done on children with hereditary hemorrhagic telangiectasia (HHT), there was a strong and reliable correlation between Epistaxis Severity Score (ESS) and Health-related Quality of Life (HR-QOL). ESS considers the frequency, duration, and intensity of bleeding episodes, as well as whether the patient is anemic, has sought medical attention, or requires a red blood cell transfusion due to nasal blood loss. Although this study was done on patients with HHT, it can be said that patients who experience severe epistaxis generally have a lower overall quality of life [23].

With regard to recurrent epistaxis impact on parents' daily life activities and quality of life, the situation is not different from what was reported for their children as most parents showed high scores for different quality of life domains, mainly communication, social functioning, and highest impact (lowest scores) was on physical and emotional functioning. The lack of severe cases, no need for medical intervention, and infrequent episodes may explain the reported low impact on children and their parents. Higher QoL was among fathers, which is logical as mothers react more to their children's health troubles, and also among parents of old age children who can afford and those with no need to have medical consolation and intervention. Other research studies reported high anxiety and affected daily life for parents of children with epistaxis, which is on the other side of the current study findings [7,9]. 

This study, while insightful, has several limitations that should be considered when interpreting its findings. One limitation is the relatively small sample size, which may not adequately represent the broader population affected by recurrent epistaxis. The limited sample size restricts the generalizability of our results, as it may not capture the full spectrum of experiences and impacts of recurrent epistaxis on children and their families. Additionally, the study's cross-sectional design limits our ability to infer causality or track changes over time, which could provide deeper insights into the condition's impact on quality of life. Future research would benefit from larger, more diverse sample sizes and longitudinal designs to more accurately assess the condition's long-term effects on family dynamics and quality of life.

## Conclusions

Our study findings indicate that recurrent epistaxis has a limited overall impact on the quality of life (QoL) of children and their parents, albeit with a moderate impact on emotional and functional domains. One notable observation is that families who manage to avoid the need for emergency interventions tend to report substantially improved QoL outcomes. This highlights the crucial role of effective management strategies for epistaxis that can mitigate its impact on family well-being. The study also reaffirms the importance of further education for parents on epistaxis management, aimed at enhancing understanding and coping mechanisms to alleviate the condition's influence on the family dynamic. Overall, despite the challenges posed by recurrent epistaxis, many families maintain a high quality of life, pointing to the resilience and adaptability of affected individuals and their support systems.
